# Nonexposure full-thickness resection in a laparoscopic-endoscopic approach as a hybrid technique for nonampullary duodenal neuroendocrine tumor

**DOI:** 10.1016/j.vgie.2025.03.032

**Published:** 2025-03-25

**Authors:** Tohru Takahashi, Yuki Miyazawa, Motoya Tominaga, Dai Miyazaki, Susumu Fukahori, Eriko Aimono, Masahiro Hagiwara, Tomoyuki Ota

**Affiliations:** 1 Department of General Surgery, Sapporo Higashi Tokushukai Hospital, Sapporo, Japan; 2 Department of Gastroenterology, Sapporo Higashi Tokushukai Hospital, Sapporo, Japan; 3 Department of Pathology, Sapporo Higashi Tokushukai Hospital, Sapporo, Japan

## Introduction

Early-stage detection of duodenal neuroendocrine tumors (d-NETs) through endoscopic screening has increased the incidence 4-fold, leading to early treatment and improved disease-specific survival.^1^ Surgical resection is recommended for d-NETs with risk of lymph node metastasis (LNM)^2^, ^3^, ^4^, ^5^, ^6^; however, d-NETs with nonfunctioning status,^7^ shallower invasion depth,^2^^,^^3^ smaller size,^3^, ^4^, ^5^, ^6^^,^^8^ and lower tumor grade^2^, ^3^, ^4^, ^5^^,^^7^ have a lower LNM risk, leading to various treatment options in the European Neuroendocrine Tumor Society guidelines.^9^

Laparoscopic-endoscopic approach as a hybrid technique, initially developed for gastrointestinal stromal tumors in the stomach,^10^ can be used for duodenal tumors to secure surgical margins and reduce delayed perforation.^11^ A conventional hybrid approach with full-thickness resection (FTR) can increase tumor dissemination risk by exposing the duodenal lumen to the abdominal cavity.^11^ Therefore, a nonexposure hybrid approach has been developed for gastric submucosal tumors.^12^ We report its application in a patient with a d-NET who underwent FTR without tumor exposure and achieved a vertical tumor-free surgical margin. This article's data will be shared by the corresponding author upon reasonable request.

## Case report

**Figure 1:**
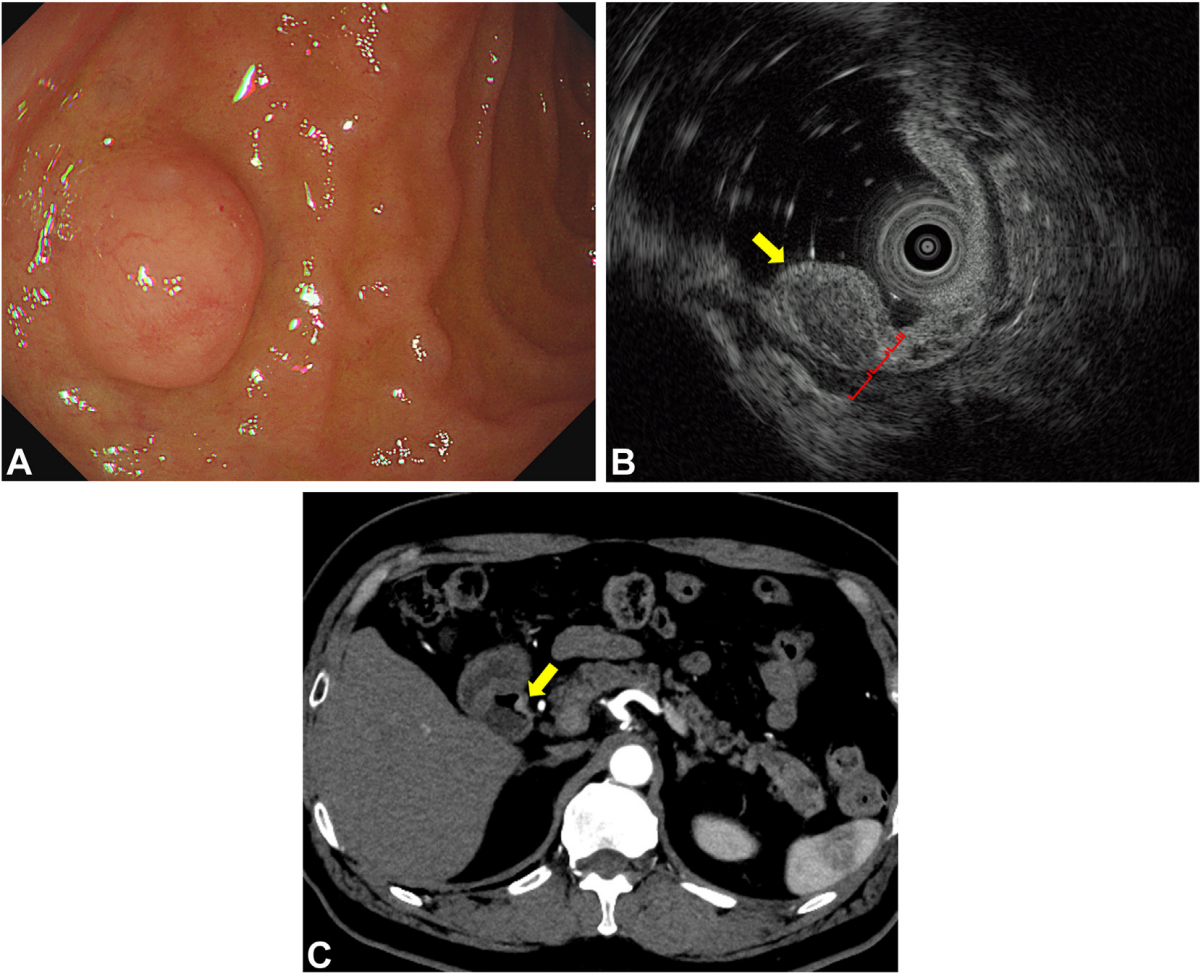
Preoperative diagnostic images. **A,** Esophagogastroduodenoscopy showing an 8-mm semicircular tumor covered with normal mucosa in the anterior wall of the duodenal bulb. **B,** Endoscopic ultrasonography identifying heterogeneous hypoechoic mass (*yellow arrow*) in the submucosal layer (third layer) without invasion into the muscular propria (fourth layer). Each layer of the duodenal wall is indicated with a square bracket. **C,** Enhanced computed tomography demonstrating a 6-mm enhanced tumor (*yellow arrow*) in the duodenal bulb without any lymphadenopathies.

The institutional ethical committee approved the publication of this report (authorization number: TGE02603-012, August 29, 2024). A 76-year-old man with a history of chronic gastritis after *Helicobacter pylori* eradication was referred to our hospital for a newly detected d-NET in the anterior wall of the duodenal bulb by regular endoscopy. Endoscopic ultrasonography identified a heterogeneous hypoechoic mass within the submucosal layer. Computed tomography revealed a 6-mm enhanced tumor without lymphadenopathies (Fig. 1). Biopsy showed an 8% Ki-67 labeling index, indicating a G2 grade. After coming to a shared decision with the patient, we performed a duodenal hybrid approach with FTR using a nonexposure technique for early-stage d-NET.

## Procedure

**Figure 2:**
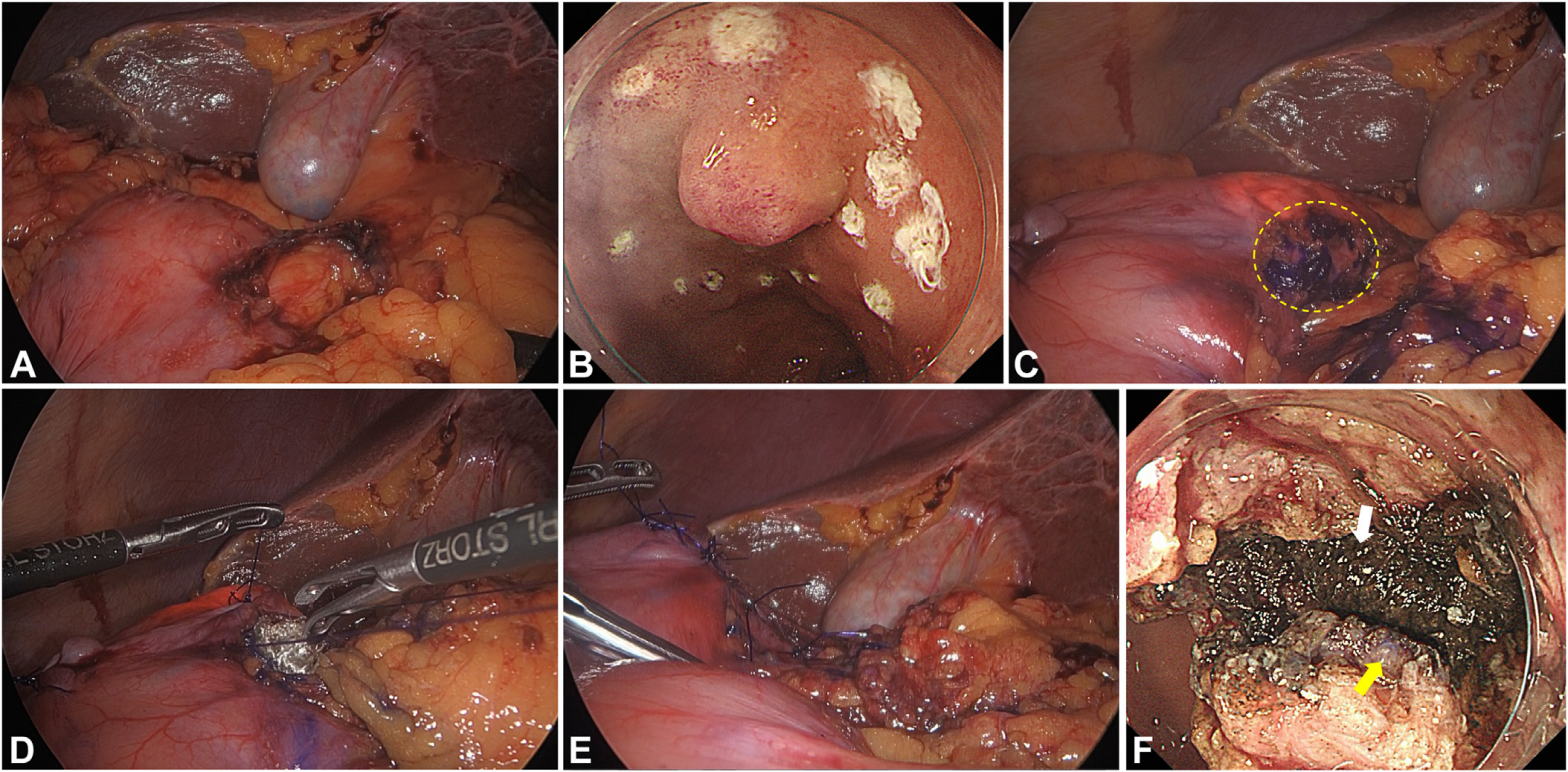
Intraoperative laparoscopic and endoscopic views. **A,** Laparoscopic mobilization of the duodenal bulb from the retroperitoneum. **B,** Endoscopic circumferential markings around the tumor with a needle-type endoscopic submucosal dissection (ESD) knife (Tech-knife; Micro-Tech Endoscopy, Ann Arbor, Mich, USA). **C,** Pigmentation of the serosal surface on the tumor with the blue dye. The *yellow dotted circle* represents the pigmented serosa. **D-E,** Placement of the hemostatic cloths (SURGICEL NU-KNIT Absorbable Hemostat; Ethicon, Raritan, NJ, USA) on the serosal surface of the tumor **(D)** followed by handsewn seromuscular suturing with 3-0 absorbable monofilament threads **(E)**. **F,** Endoscopic full-thickness resection with the ESD knife showing the pigmented serosa (*yellow arrow*) with the hemostatic cloths (*white arrow*).

With the patient under general anesthesia, the right colic flexure and duodenal bulb were mobilized from the retroperitoneum using a laparoscopic procedure (Fig. 2A). Endoscopic peritumoral markings were placed circumferentially with a needle-type endoscopic submucosal dissection (ESD) knife (Tech-knife; Micro-Tech Endoscopy, Ann Arbor, Mich, USA) (Fig. 2B).

The tumor serosa was laparoscopically marked with blue dye and covered with hemostatic cloths (SURGICEL NU–KNIT Absorbable Hemostat; Ethicon, Raritan, NJ, USA), functioning as spacers, followed by handsewn seromuscular suturing (Fig. 2C-E). Endoscopic FTR was performed using the ESD knife after identifying the pigmented serosa and hemostatic cloths (Video 1, available online at www.videogie.org, Fig. 2F). The operative time was 221 minutes. Remarkably, the patient received no painkillers and was discharged on postoperative day 8 without any adverse events.

**Figure 3:**
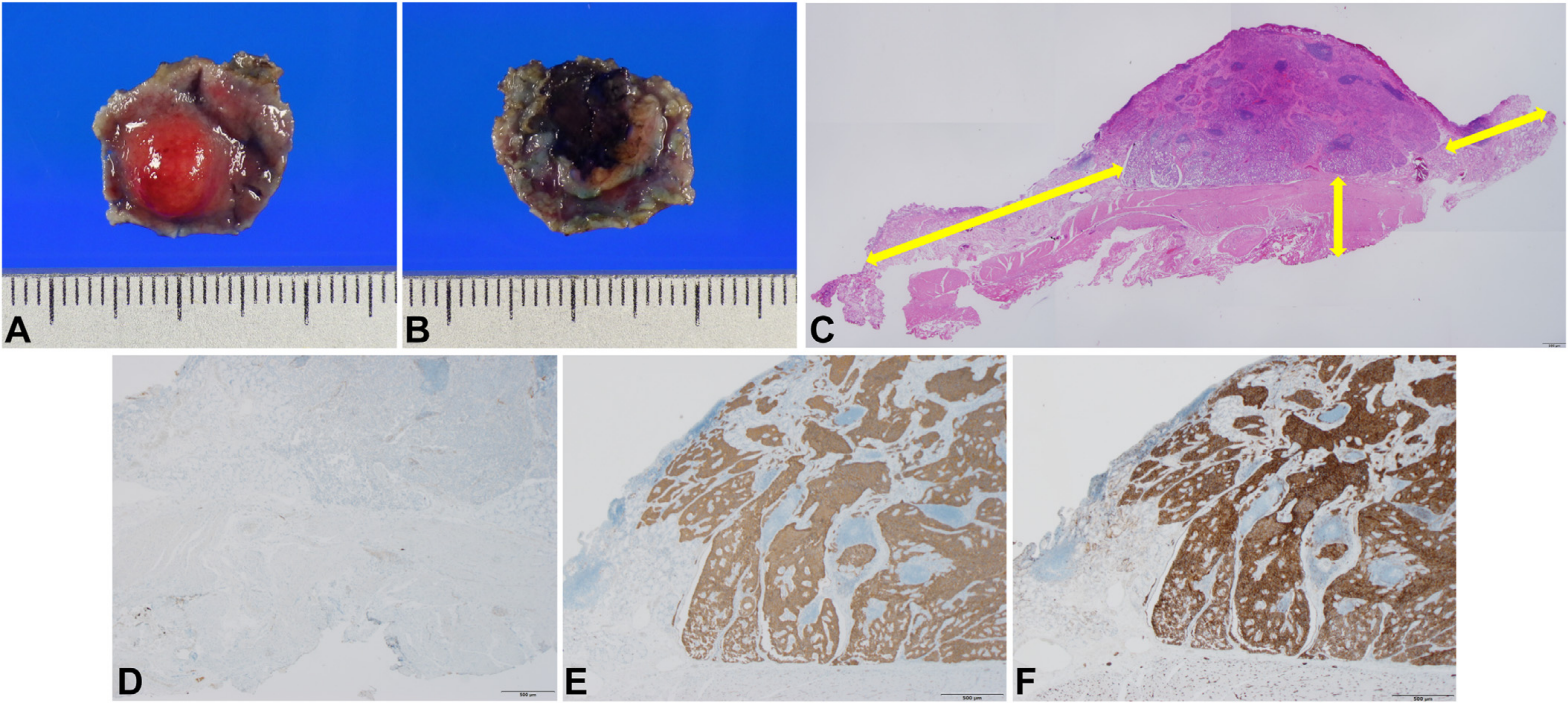
Macroscopic images and histopathologic examinations of the resected duodenal tumor. **A-B,** The resected specimen showed that the duodenal tumor (8 × 8 mm) was covered by the normal mucosa **(A)** and serosa **(B)** indicating a negative vertical margin. **C,** Hematoxylin and eosin staining identified the tumor in the deeper lamia propria invading the submucosal layer, although lymphovascular invasion was not observed (×12.5). The tumor was removed with full thickness in sufficient horizontal and vertical margins (*double yellow arrows*). **D-F,** Immunohistochemical finding revealed positive staining for D2-40 **(D)** in the serosal mesothelium beneath the resected tumor (×20) and confirmed the tumor as a neuroendocrine tumor with positive staining for synaptophysin **(E)** and CD56 **(F)** (×20). The scale bars represent 500 μm **(C-F)**.

Histopathology revealed an 8-mm tumor in the lamina propria, and the serosal mesothelium beneath it was D2-40 positive, verifying the negative vertical margin (Fig. 3A-D). Immunohistochemistry showed positive staining for synaptophysin and CK56, with <1% Ki-67 labeling index confirming G1 grade (Fig. 3E-F). Follow-up computed tomography showed no recurrence for 6 months postoperatively.

## Discussion

The duodenal hybrid approach, involving whole-layer or seromuscular suturing, achieves a greater complete resection rate (95.1%) with reduced intraoperative and delayed perforation (4.9% and 2.4%, respectively)^11^ than ESD (29%-90%, 19%-35%, and 3%-20%, respectively) for superficial nonampullary duodenal epithelial tumors.^13^ Band ligation without resection is a safe, noninvasive alternative that provides a shorter hospital stay and minimal risk of adverse events, including bleeding, perforation, and stricture, making it suitable for smaller d-NETs.^14^^,^^15^ It carries the risk of residual tumor cells and recurrence, as vertical margins and tumor grading cannot be evaluated without a resected specimen.^15^ Frequent follow-up endoscopy with biopsy is required, and patients may lose the opportunity for further standard surgery.

**Figure 4:**
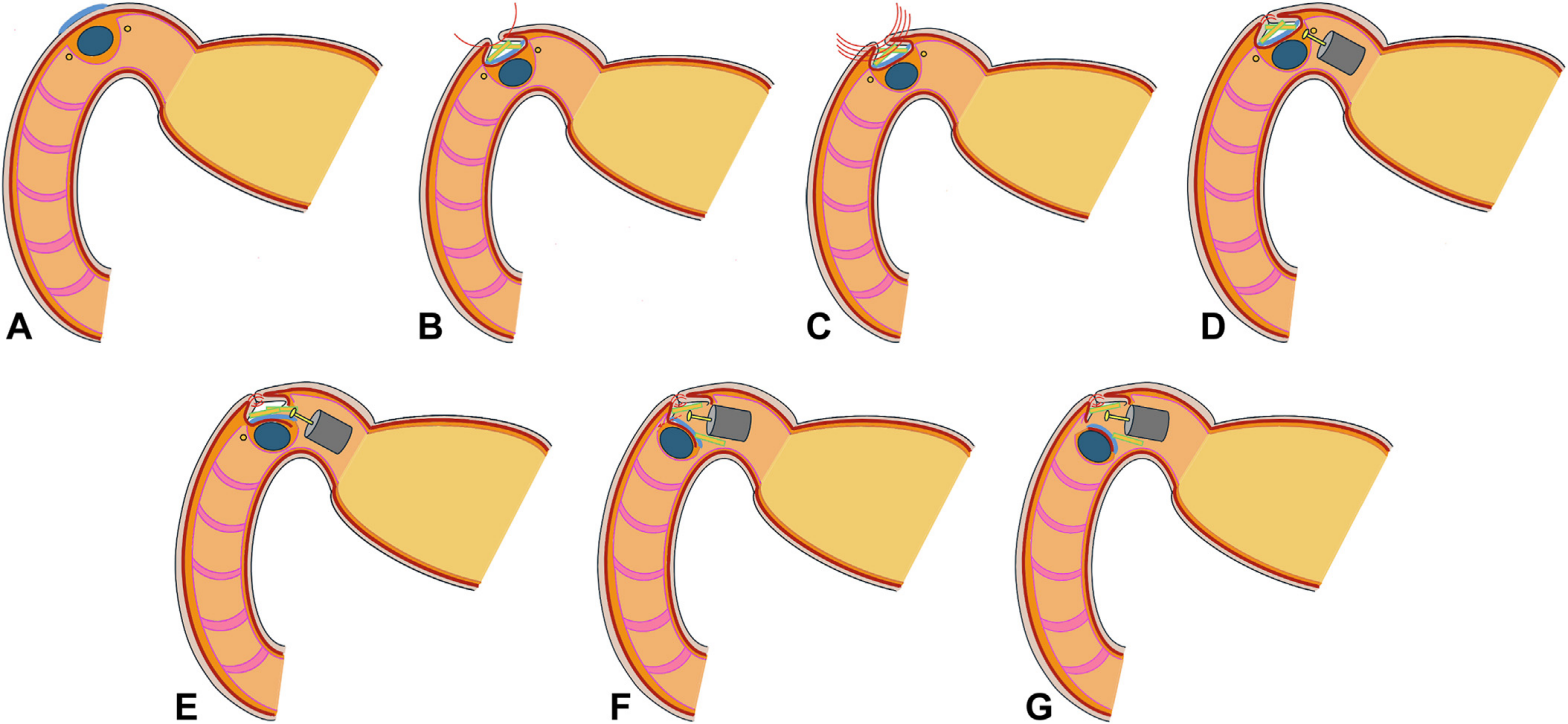
Description of technique. **A,** Serosal pigmentation beneath the tumor. **B-C,** Mounting the hemostatic cloths as spacers on the pigmented serosal surface followed by seromuscular suturing to bury them. **D-F,** Endoscopic full-thickness resection was performed by cutting each layer one by one, and recognition of pigmented serosa and spacers prevented the ESD knife from moving forward to cut the seromuscular sutured threads or injure the covered serosa. **G,** Removal of the tumor from the luminal side.

Several nonexposure techniques have been developed to prevent tumor cell spread and an inaccurate vertical margin at the cutting border due to thermal injury.^12^^,^^16^^,^^17^ However, they can cause intraoperative perforation^16^ or stapler stricture.^17^ Our procedure benefits from using hemostatic cloths as spacers, which absorb bloody effusion and help maintain clear vision during FTR with an ESD knife. We pigmented the serosa to identify the cutting layer from the luminal side. Notably, pigmentation with spacers helps prevent the knife from moving forward extensively, avoiding cutting the threads or injuring the covered serosa (Fig. 4).

Abe et al^18^ first reported laparoscopy-assisted endoscopic FTR using the ESD technique for a duodenal carcinoid tumor with an open approach. In contrast, Ohata et al^19^ performed endoscopy-assisted laparoscopic FTR openly and subsequently altered it to laparoscopy-assisted endoscopic FTR with ligation and snare technique for nonampullary duodenal neoplasms in a closed manner.^20^ Although our endoscopic procedure is similar to that described in the report from Abe et al, we performed laparoscopic seromuscular suturing beforehand to prevent tumor-cell dissemination, as in Ohata et al’s technique.^20^ Considering the tumor size, our endoscopic FTR technique provides a broader indication for duodenal tumors than the ligation and snare technique.^20^

This study has some limitations. Long-term oncologic outcomes could not be assessed because of the absence of lymph node dissection, which requires dissection around the pancreas and can lead to invasiveness and severe adverse events. The United States Neuroendocrine Tumor Study Group identified LNM in 40% of d-NETs <1 cm and required minimal retrieval of 8 lymph nodes for accurate staging.^21^ Tumor location also influences the difficulty of seromuscular handsewn suturing, particularly for periampullary tumors.

In conclusion, FTR with a nonexposure technique in a duodenal hybrid approach is a feasible minimally invasive method to avoid tumor-cell dissemination and delayed perforation with accurate resectability for patients with d-NETs.

## Patient consent

The patient in this article has given written informed consent to publication of the case details.

## Disclosure

All authors disclosed no financial relationships.

## Supplementary data

Video 1 Nonexposure full-thickness resection in laparoscopic-endoscopic approach as a hybrid technique for nonampullary duodenal neuroendocrine tumor.
